# Angiotensin II in ECMO patients: a word of caution

**DOI:** 10.1186/s13054-019-2337-5

**Published:** 2019-04-26

**Authors:** Elio Antonucci, Fabio Silvio Taccone

**Affiliations:** 1 Intermediate Care Unit - Emergency Department - Ospedale Guglielmo da Saliceto, Piacenza, Italy; 2 Department of Intensive Care, Laboratoire de Recherche Experimentale, Erasme Hospital, Brussels, Belgium

We read with interest the recent letter by Ostermann et al. [1] about the administration of angiotensin II (Ang II) in patients treated by extracorporeal membrane oxygenation (ECMO). The authors reported seven patients with severe cardiorespiratory or respiratory failure receiving Ang II infusion during ECMO in the setting of high catecholamine doses. After Ang II initiation, they found a significant increase in blood pressure and a quick reduction in the need of other vasopressors. Despite the novelty of these findings, a word of caution should also be considered.

First, two patients were treated with veno-venous (VV) ECMO, that is often initiated in the case of severe acute respiratory distress syndrome (ARDS). Renin-angiotensin system (RAS) has been implicated in the pathogenesis of ARDS. In particular, Ang II is a pro-inflammatory molecule able to promote capillary leak and fibroproliferation. Also, Ang II may contribute to a significant constriction of the pulmonary circulation, potentially leading to reduced blood flow and to increased ventilation/perfusion mismatch [2]. Opposite to the angiotensin-converting enzyme (ACE)/Ang II axis, RAS has a counter-regulatory axis, which is composed by angiotensin-(1–7) (Ang 1–7) and ACE2 [2]. Since ACE2 can minimize the risk of acute lung injury, a recombinant form of human ACE2 (rhACE2) has been tested in patients with ARDS in a recent phase II trial [2]; this study showed that rhACE2 increases surfactant protein D concentrations without major side effects. Also, in an experimental model of ARDS, Ang 1–7 led to a significant improvement in oxygenation, decreasing the severity of acute lung injury and inflammation [3].

Second, five patients were treated with veno-arterial (VA) ECMO, that is usually implemented in cases of severe cardiac failure. No information on cardiac function was provided in this series, while the safety profile of Ang II has never been tested in patients with vasodilatory shock and severe concomitant myocardial dysfunction. Indeed, Ang II could reduce the cardiac output due to its vasoconstrictive properties and provide some detrimental effects on myocardial recovery [4].

Finally, ECMO patients need an adequate accurate anti-coagulation for the integrity of the extracorporeal system. Ang II usually exerts pro-coagulant effects, stimulating thrombin generation and potentially damaging microcirculation [5]. For all these reasons, we think that more safety and physiological data on the effects of Ang II should be collected before to extend its use in ECMO patients.

## Abbreviations

ACE: Angiotensin-converting enzyme
Ang 1–7: Angiotensin 1–7
Ang II: Angiotensin II
ARDS: Acute respiratory distress syndrome
ECMO: Extracorporeal membrane oxygenation
RAS: Renin-angiotensin system
rhACE2: Recombinant form of human ACE2
VA: Veno-arterial
VV: Veno-venous
