# Supplementary material for: Angiotensin II in ECMO patients: a word of caution
Source: Crit Care. 2019 Apr 26;23:144. doi: 10.1186/s13054-019-2337-5 (PMC6485103; doi:10.1186/s13054-019-2337-5)
Supplement: Related texts

# Reply: Authors’ response

**Authors:** Marlies Ostermann, Kyle Gunnerson

We appreciate the comments of Professors Antunucci and Taccone who acknowledge the hemodynamic improvement associated with the use of angiotensin II (Ang II) in patients on ECMO requiring high-dose vasopressors but offer a word of caution related to ARDS and cardiogenic shock.

With regard to ARDS, animal research has suggested that Ang II is detrimental in ARDS, hence the clinical development of recombinant ACE-2 (rhACE-2), an enzyme that converts Ang II into Ang 1-7. Studies in animals were promising. However, the clinical trial by Khan et al which Professors Antunucci and Taccone refer to, tested rhACE-2 in patients with ARDS and failed to show an improvement in oxygenation or any physiological parameters [2]. In fact, the trial was terminated early following a futility analysis. Interestingly, this study also showed that 71% of patients had low baseline concentrations of Ang II <50pg/ml. This is in keeping with the results by Orfanos et al who demonstrated that severe ARDS was associated with angiotensin converting enzyme dysfunction resulting in Ang II deficiency [6]. Lastly, we would argue that a theoretical improvement in oxygenation, as suggested by animal studies, is less relevant during ECMO when oxygenation is provided via the extracorporeal circuit.

The authors’ second point is that Ang II may decrease cardiac output. Whilst this is in theory possible, previous studies have shown that administration of bovine angiotensin II successfully rescued patients with cardiogenic shock, including patients in a cardiac arrest situation [7]. We would also like to point out that Ang I is largely converted to Ang II in the lungs. During VA ECMO, a significant proportion of blood is drawn via the venous cannula into the ECMO circuit and bypasses the lung. This is likely to contribute to Ang II deficiency and supports the case for Ang II in this setting [1].

Finally, Ang 1-7 is a potent vasodilator with effects opposite those of Ang II. Ang 1-7 builds up when ACE is not functional. The administration of Ang II may decrease Ang 1-7 via biofeedback resulting in an increase of blood pressure and decrease of the catecholamine dose [8].

We fully agree that more research is needed to determine the role of Ang II in patients on ECMO. Given that the bovine form of angiotensin II was utilized safely and effectively for over 45 years to treat shock, data in the literature may help us developing thoughtful prospective trials.
